# Decoding identity-related expression in Chinese vocal music using computational acoustic and lyric features

**DOI:** 10.3389/fpsyg.2026.1856765

**Published:** 2026-07-15

**Authors:** Xiaoyu Wang

**Affiliations:** Arts College, Northeastern University of China, Shenyang, Liaoning, China

**Keywords:** acoustic study, Chinese music, computational musicology, language, national identity

## Abstract

Vocal performance provides a measurable interface between linguistic structure and musical expression, making it a tractable domain for investigating how culturally meaningful features are encoded in sound. In Chinese vocal music, this interface is particularly salient due to the interaction between tonal language systems and melodic organization, raising the question of how identity-related expression may be distributed across textual and acoustic dimensions. This Registered Report proposes a multimodal computational framework to examine identity-related expression in Chinese vocal music by integrating lyrics, speech, and singing data. A three-layer corpus will be constructed based on vocal works identified in recent Chinese core-journal literature on music and identity. Natural language processing (NLP) will be used to extract high-frequency and sentiment-bearing lyric units, which define preregistered anchor points for subsequent speech recording and audio segmentation. Acoustic features—including pitch (F0), intensity, and contour-based descriptors—will be extracted from both sung and spoken realizations of matched lyric units. Cross-modal relational features will be computed to quantify correspondence between speech and singing. This protocol specifies confirmatory analyses to examine how textual, acoustic, and cross-modal features relate to independently collected identity ratings, using correlation analysis, principal component analysis (PCA), and Random Forest Regression (RFR). Model performance will be evaluated via cross-validation, and feature importance will be used to compare the relative contributions of linguistic and musical dimensions. The hypotheses, preprocessing steps, and analytical procedures are specified in this Stage 1 protocol to ensure transparency and reproducibility. A pilot analysis conducted on a representative excerpt demonstrates that the proposed pipeline can reliably extract comparable acoustic features across modalities, supporting the feasibility of the preregistered design. By formalizing a computational pathway linking vocal performance features to identity-related expression, this study aims to contribute to performance science and computational musicology by providing a replicable framework for analyzing culturally embedded vocal expression.

## 1 Introduction

Vocal performance is a form of human performance in which embodied vocal technique (breath management, phonation, resonance shaping, articulation) is continuously coordinated with culturally learned expressive choices (text delivery, melodic shaping, timing, and vocal colour). This makes singing a tractable object for Performance Science because it links observable performance behaviours to psychological functions (e.g., expression and communication) and to socially meaningful outcomes. Across music scholarship, songs are commonly treated as culturally embedded artefacts whose meanings and uses are inseparable from social relations and political life, motivating analyses that connect musical practice to the politics of culture and to the sociology of music (Ivo, 2005; Norris, 1989). Within this perspective, lyrics and vocal works can function as ideological carriers and as symbolic resources through which values become communicable and contestable in public discourse (Williams, 2016). In contemporary discussions of Chinese nationalism and national identity, scholars have highlighted both the centrality of identity narratives and the need for more precise, empirically grounded characterisations of the phenomena (Carlson, 2009; Carrico, 2023). Work on centennial “red songs” suggests that politically salient vocal repertoires can organise collective memory and emotional experience through structured lyric narratives and their circulation (Zheng, 2021). Building on these foundations, this Stage 1 Registered Report frames vocal art as a performance site where identity-related expression may be realised through jointly patterned lyrical and acoustic choices, and it specifies pre-registered questions and predictions about how such patterns can be operationalised and tested.

Chinese vocal music is particularly well suited for investigating the speech–melody interface because it sits at the intersection of linguistic pitch organisation and musical pitch organisation. Many Chinese languages are tonal, meaning that pitch patterns contribute to lexical contrasts; this immediately raises a measurable question for sung performance: how are speech-based pitch constraints accommodated, preserved, or reshaped when syllables are mapped onto melodic contours? Corpus-based work on Chaozhou songs—a Southern Chinese dialect with a complex tonal system—demonstrates that tone–melody correspondence can be analysed quantitatively and that contextual tonal processes (e.g., tone sandhi) matter for the mapping between linguistic tone and musical pitch (Zhang and Cross, 2021). More broadly, language–music scholarship in anthropology argues that the boundary between “language” and “music” is not given in nature but is culturally constructed, and that sound and text can be studied as a unified semiotic field (Faudree, 2012). This view aligns with sociological accounts that treat musical systems as historically rationalised and socially grounded, including analyses that relate musical organisation to broader cultural conditions (Weber, 1958). In China, debates on the mutual constitution of “music in language” and “language in music” underscore the analytical relevance of treating vocal works as joint music–text systems (Wang and Su, 2022). Finally, because national language policies shape which varieties gain authority and circulation, Chinese vocal repertoires also provide a context in which identity-related expression may be linked to language standardisation and dissemination (Jie, 2015). In this Registered Report, these properties motivate a syllable/word-level pairing design in which selected lyric units are aligned to both spoken renditions and sung realisations, enabling pre-registered, acoustically grounded comparisons of pitch contours, ranges, and cross-modal correspondence metrics.

To study identity-related expression as a performance phenomenon, a purely interpretive reading of lyrics or a purely acoustic description of singing is insufficient: identity cues may be distributed across semantic content, selection of topics and evaluative language, and the fine-grained vocal realisation of those texts in performance. Accordingly, this Stage 1 Registered Report proposes a multimodal computational framework that makes the inferential pathway explicit and preregistrable. The planned corpus is three-layered—lyrics text, sung audio, and recorded spoken renditions of matched lyric units—so that each analytical unit can be compared within and across modalities. On the lyrics side, natural language processing (NLP) will be used to extract high-frequency words and sentiment-bearing words, which serve as theoretically motivated “anchors” for sampling: they define which lyric tokens/phrases are targeted for subsequent melody-segment selection and speech recording, thereby reducing researcher degrees of freedom in corpus segmentation. This strategy is directly motivated by computational modelling of Chinese songs in which word-count–based lyric features (including emotion-related word frequencies) were linked to musical constructs and integrated with audio features in machine-learning models (Xu et al., 2024). On the performance side, acoustic feature extraction will target low-level parameters (e.g., F0-related measures, intensity), higher-level prosodic/structural descriptors (e.g., contour shape, local variability), and—crucially—cross-modal relational features that quantify the correspondence or divergence between spoken and sung realisations for the same lexical items. Prior comparative music–phonetics work on Chinese art songs shows the feasibility of representing speech-sound aspects embedded in vocal performance using frequency, intensity, and pitch measures, supporting the validity of the planned speech–singing pairing logic (Xiaoyu et al., 2024). The analysis plan specified in this protocol tests these predictions through correlation analyses (association), principal component analysis (dimensional structure), and Random Forest Regression (nonlinear modelling and feature-importance ranking), with clear separation of confirmatory and exploratory components as required for Registered Reports.

### 1.1 The present study

Building on the theoretical and empirical gaps identified above, the present study aims to systematically examine how national identity is embedded and articulated in Chinese vocal art through the interaction of musical and linguistic dimensions. To address this objective, the study specifies three confirmatory hypotheses. H1: Lyric features derived from high-frequency content words and sentiment-bearing words are expected to provide measurable cues for identity-related expression in Chinese vocal music. H2: Acoustic and prosodic features extracted from spoken and sung realizations of matched lyric units are expected to capture additional aspects of identity-related expression beyond lyric content alone. H3: Cross-modal relational features comparing speech and singing are expected to contribute to the characterization of identity-related expression by indicating how linguistic and musical dimensions are coordinated within vocal performance.

These hypotheses will be examined through a quantitative, corpus-based design grounded in a multidimensional linguistic–musicological approach. The following Methods section details the corpus construction, feature extraction procedures, and analytical strategies employed to implement this design. A pilot analysis conducted on the phrase “我爱你中国 (I Love You, China)” demonstrates that the proposed pipeline can successfully extract comparable acoustic features from both speech and singing, supporting the feasibility of the preregistered design.

## 2 Method

### 2.1 Multimodal corpus construction

**Figure 1:**
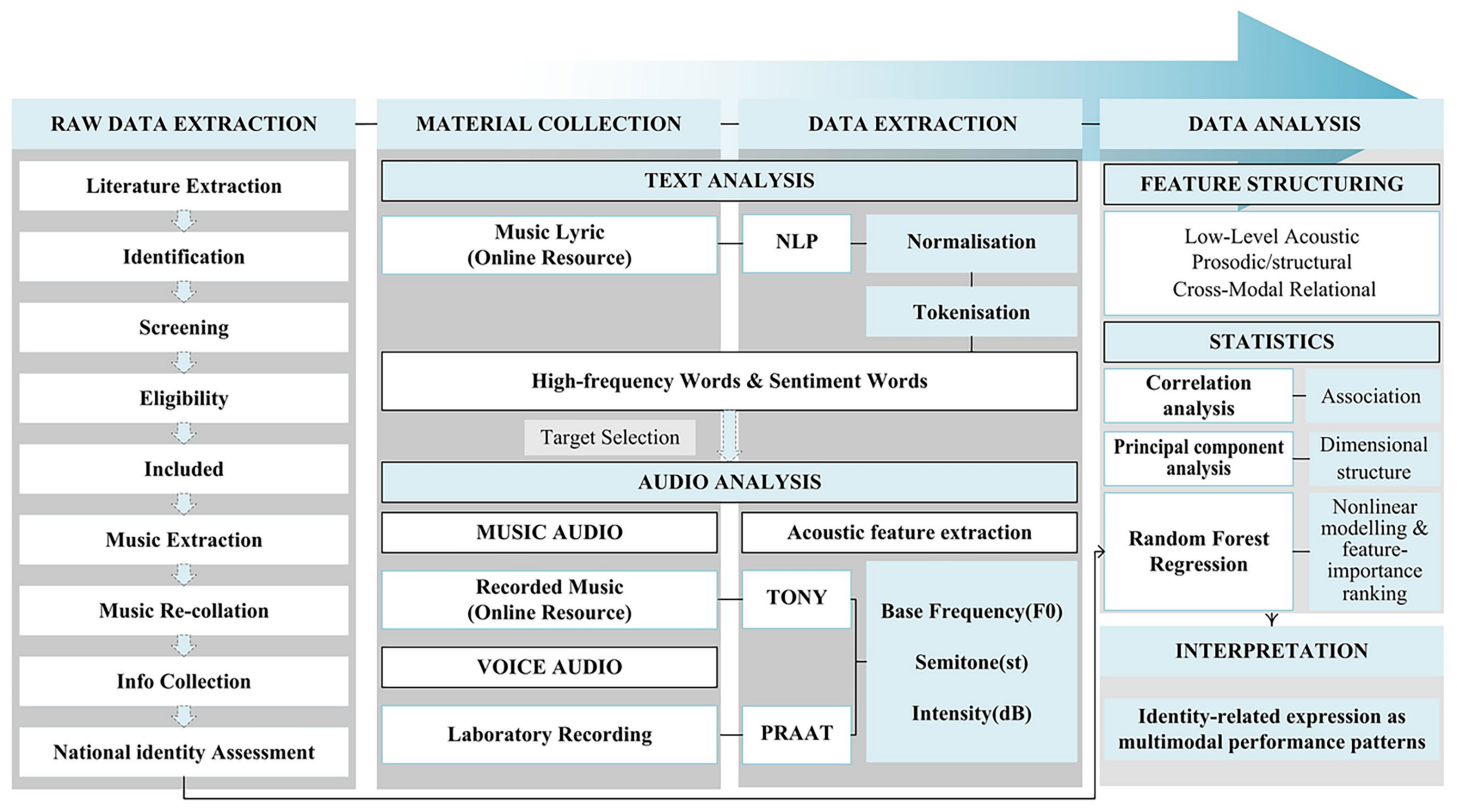
Flow chart of the present study.

This Stage 1 Registered Report preregisters a three-layer multimodal corpus comprising (i) lyrics text, (ii) sung audio (song recordings), and (iii) matched spoken renditions (speech recordings) for selected lyric units. The pool of target songs (genre: Chinese vocal music / song) is derived via a literature-to-repertoire approach. We will conduct a literature search on CNKI^1^ using the Chinese keywords “音乐 AND 认同” (“music and identity”) and “中国性 AND 音乐” (“Chinese characteristics and music”). Inclusion criteria are: (a) publication year 2020–present, and (b) core-journal indexing categories SCI, CSSCI, AMI, CSCD, EI, and PKU Core. From each included article, we will extract all explicitly analysed or exemplified vocal works used as instances of national identity/“Chinese characteristics” claims, then deduplicate titles to form the final song list. Based on the scale of comparable computational music studies, including cross-cultural analyses using 304 recordings (Savage et al., 2015) and computational modelling of emotional responses using 222 pieces of music (Mori, 2022), we expect the present corpus to contain approximately 200–300 Chinese vocal works. If the final eligible corpus falls below the minimal threshold (n < 200), the reason will be documented in the Stage 2 manuscript together with the full selection record and metadata table (see Figure 1).

For each song, we will obtain the complete lyrics and at least one usable recording. Following common practice in computational music research(Chu et al., 2020; Wang et al., 2022), lyrics and recordings will be collected from publicly accessible, legally traceable, or licensed sources, including official publications, public music platforms, archives, and authorised recordings. If a selected work is copyrighted, it will be used only when lawful access or permission is available; otherwise, it will be excluded from the audio-analysis and listening-rating components. Original copyrighted lyrics and audio files will not be redistributed, and only metadata, derived lyric/acoustic features, annotations where legally shareable, and analysis code will be made available at Stage 2.

If multiple recordings are available, the priority rule is to select a version that (i) contains clear vocals suitable for stable F0 and intensity extraction, and (ii) matches the published lyrics with minimal mismatch. For recording selection, the author will first generate a candidate list according to predefined inclusion and exclusion criteria: vocal clarity sufficient for F0 and intensity extraction, absence of severe distortion or clipping, and close correspondence between the recording and the verified lyrics. Two musicology experts independent of the study will then evaluate the candidate recordings separately. Disagreements will be resolved through discussion, and the final selected version for each song will be determined by consensus and documented in the metadata.

Speech recordings will be collected from two native Mandarin speakers (one male, one female; coded M and F), both from an official Northern Mandarin dialect area and having passed the official Mandarin proficiency test (Level 1). Prior to recording, three professors specialising in Chinese linguistics will judge the speakers as natural and representative of Mandarin and cultural tradition. Speech recordings will follow predefined procedures for hardware/software specifications, laboratory environment, noise control, microphone distance, sampling format, practice reading, and re-recording criteria.

### 2.2 Identity assessment procedure

To obtain an external criterion for identity-related expression at the excerpt/song level, we will deploy an online listening-and-rating study using an internal web platform (Flask backend; Tomcat server; HTML/JavaScript front-end) that implements a multi-step procedure (consent → randomised audio presentation → questionnaire submission). Participants will be Chinese adults who self-report no hearing impairment and complete all listening tasks. Identity ratings will use a 5-item, 5-point Likert Identity Assessment Scale (IAS; 1 = strongly disagree to 5 = strongly agree), adapted from the national identity dimension in Chunling and Senlin (2020) and supported by validation evidence reported in Qu et al. (2021).

The minimum sample size was estimated using the Normal Approximation method in PASS (Power Analysis and Sample Size Software, 2017, NCSS, Kaysville, Utah, USA, ncss.com/software/pass). Following prior recommendations for reliability-based sample-size planning, the expected reliability level was set at *α* = 0.909 based on previous national identity scale validation, and the minimum acceptable reliability level was set at α = 0.70. With an alpha risk of 5%, beta risk of 10%, an assumed difference of 0.209, and an anticipated dropout rate of 20%, a minimum of 33 valid participants per condition is required. In the present design, identity ratings will be collected across four listening conditions: singing, speech, melody/instrumental, and pink-noise control. Therefore, the minimum total target is 132 valid responses. To ensure adequate data quality for the planned predictive modelling, we will aim to recruit at least 160 participants and will stop data collection only after this minimum target has been met.

### 2.3 Feature extraction procedure

Feature extraction begins with lyrics-based NLP preprocessing. Text normalisation (removing non-lyric annotations and repeated structural markers), Chinese word segmentation using jieba, stopword removal, and computation of term frequency and TF–IDF. Anchor selection is defined as follows: the top 10% of words by frequency (computed over the full lyrics corpus) will be selected as high-frequency anchors. In parallel, sentiment-bearing words will be identified using the Dalian University of Technology Chinese Sentiment Lexicon Ontology, a publicly available Chinese sentiment resource developed for sentiment analysis and opinion mining (Xu et al., 2024). The lexicon contains 27,466 words annotated with part of speech, emotion category, intensity, and polarity, covering seven major emotion classes and 21 subcategories. In the present study, it is used to identify sentiment-bearing lyric units, which form the second anchor set for subsequent audio alignment, slicing, and speech recording prompts. The union of (high-frequency anchors + sentiment words) defines the target list for (i) audio alignment and slicing and (ii) speech recording prompts.

Word-level lyrics–audio alignment and segment slicing will use a semi-automatic forced-alignment workflow centred on Montreal Forced Aligner (MFA). First, recordings will be preprocessed; when needed, we will apply a vocal separation step to reduce accompaniment interference (e.g., https://vocalremover.org/zh/), then convert to a standard audio format (mono, 41 kHz sampling rate). Second, lyrics anchors will be normalised and converted to tone-marked pinyin sequences (e.g., via pypinyin) with a preregistered tokenisation granularity (character-level or word-level) chosen to preserve a one-to-one mapping structure. Third, we will build an MFA-compatible corpus folder (wav + corresponding.txt pinyin) and run alignment using a preregistered Chinese acoustic model and pronunciation dictionary, producing TextGrid outputs with word and phone tiers. Fourth, because singing acoustics often deviate from conversational speech (e.g., vowel lengthening, legato, ornamentation), we will manually correct TextGrid boundaries in Praat by inspecting waveform and spectrogram cues (onset, steady-state, offset), using phone-tier information to refine word boundaries when needed. Fifth, corrected TextGrids will be programmatically parsed (Python) to slice the vocal track into word-level audio segments and export consistent filenames (token + timestamps). In melismatic cases (one character spanning multiple notes/segments), we will retain the full token span and compute duration-weighted aggregations for note-level measures to preserve comparability.

Acoustic feature extraction follows priori settings specified for both modalities. For singing segments, we will extract F0 trajectories and note-related information using Tony with the pYIN algorithm; for speech segments we will extract F0 using pYIN as well, to maximise methodological consistency. We will also extract intensity and duration measures (procedure/software to be preregistered consistently across modalities). All F0 values (Hz) will be converted to semitones (st) relative to A4 = 440 Hz using a conversion rule. We will report handling of unvoiced frames, smoothing, and missing values.

Features will be organised into three preregistered categories:

(1) Low-level acoustic features: F0 mean/median, SD, range, interquartile range; intensity mean/range; token duration.
(2) Prosodic/structural features: contour slope (linear fit of F0 over time), onset–offset difference, local peak counts/positions (as preregistered).
(3) Cross-modal relational features (speech–singing pairing): mean pitch difference (singing minus speech), range ratio (singing range / speech range), and contour similarity. For contour similarity, we will compute similarity on each paired token by interpolating each F0 contour onto a common within-token time grid (interpolation method and number of preregistered points) and then computing a preregistered similarity metric (e.g., correlation). We will not perform across-dataset duration normalisation for pooling; comparisons will focus on within-corpus contrasts between predefined token classes (high-frequency vs. sentiment anchors) and between modalities, while contour similarity is computed within each paired token.

### 2.4 Analytical strategies

Analyses listed below are confirmatory unless explicitly labelled exploratory; any deviations or additional analyses at Stage 2 will be reported as exploratory.

*Outcomes*. The primary criterion outcome is IAS-derived identity rating (aggregated at preregistered level: excerpt-level and/or song-level mean). Secondary outcomes include preregistered text-based indices derived from anchor distributions (e.g., normalised frequencies and sentiment-category proportions). Predictor sets comprise (a) lyrics NLP features, (b) speech acoustic/prosodic features, (c) singing acoustic/prosodic features, and (d) cross-modal relational features.

*Correlation analyses*. We will test the associations among (i) lyrics indices, (ii) acoustic/prosodic features, (iii) cross-modal relational features, and (iv) IAS ratings. We will preregister correlation type (Pearson), alpha level, effect-size reporting, and multiple-comparison control (e.g., FDR). Where appropriate, analyses will be stratified by anchor class (high-frequency vs. sentiment) and modality.

*Principal component analysis (PCA)*. To characterise shared structure in the multimodal feature space, PCA will be conducted on standardised feature matrices using predefined retention (e.g., eigenvalue criterion plus scree/parallel criterion), rotation method (if any), and interpretation criteria (loading thresholds). PCA is intended for dimensional interpretation and as a structured input summary, not for data-driven *post hoc* feature selection.

*Random Forest Regression (RFR)*. We will use RFR to model potentially nonlinear mappings from features to IAS ratings and to compare the predictive contribution of modality-specific vs. combined predictors, consistent with prior computational modelling linking lyric-derived and audio-derived features in Chinese songs (Xu et al., 2024). We will fit three models: (1) language-side model (lyrics NLP + speech features), (2) music-side model (singing features), and (3) combined multimodal model (lyrics + speech + singing + cross-modal relational features). Hyperparameters will be tuned via the grid-search procedure, evaluated by 10-fold cross-validation, and reported using *R*^2^ and RMSE. Feature importance will be reported using Gini (impurity-based) importance, with a caution about known biases and a sensitivity analysis using permutation importance (Strobl et al., 2009). Comparative interpretation will focus on relative rankings and cross-validated performance differences, not on absolute importance magnitudes. The inclusion of explicit speech–singing relational predictors is motivated by the feasibility of comparative music–phonetics operationalisation in Chinese art-song contexts (Xiaoyu et al., 2024).

## Data Availability

The original contributions presented in the study are included in the article/supplementary material, further inquiries can be directed to the corresponding author.

## Footnotes

1
www.CNKI.net
